# Manipulating motor performance and memory through real-time fMRI neurofeedback

**DOI:** 10.1016/j.biopsycho.2015.03.009

**Published:** 2015-05

**Authors:** Frank Scharnowski, Ralf Veit, Regine Zopf, Petra Studer, Simon Bock, Jörn Diedrichsen, Rainer Goebel, Klaus Mathiak, Niels Birbaumer, Nikolaus Weiskopf

**Affiliations:** a Department of Radiology and Medical Informatics—CIBM, University of Geneva, Rue Gabrielle-Perret-G 4, CH-1211 Geneva 14, Switzerland; b Institute of Bioengineering, Swiss Institute of Technology Lausanne (EPFL), STI-IBI Station 17, CH-1015 Lausanne, Switzerland; c Institute of Medical Psychology and Behavioral Neurobiology, University of Tübingen, Gartenstrasse 29, 72074 Tübingen, Germany; d Perception in Action Research Centre, ARC Centre of Excellence in Cognition and its Disorders, Department of Cognitive Science, Macquarie University, Sydney 2109, NSW, Australia; e Department of Child & Adolescent Mental Health, University Hospital of Erlangen, Schwabachanlage 6+10, 91054 Erlangen, Germany; f Department of Child & Adolescent Psychiatry and Psychotherapy, Centre for Mental Health, Hospitals of Stuttgart, Prießnitzweg 24, 70374 Stuttgart; g Institute of Cognitive Neuroscience, University College London, 17 Queen Square, London WC1 N 3AR, UK; h Department of Cognitive Neuroscience, Maastricht University, Maastricht 6200 MD, The Netherlands; i Netherlands Institute for Neuroscience, Royal Netherlands Academy of Arts and Sciences (KNAW), 1105 BA Amsterdam, The Netherlands; j Department of Psychiatry, Psychotherapy and Psychosomatics, RWTH Aachen University, Aachen, Germany; k Ospedale San Camillo, Istituto di Ricovero e Cura a Carattere Scientifico, Venezia-Lido, Italy; l Wellcome Trust Centre for Neuroimaging, UCL Institute of Neurology, University College London, 12 Queen Square, London WC1 N 3BG, UK

**Keywords:** Memory, Motor performance, Neurofeedback, Brain imaging, Functional magnetic resonance imaging (fMRI), Real-time fMRI, Self-regulation, Brain training

## Abstract

Task performance depends on ongoing brain activity which can be influenced by attention, arousal, or motivation. However, such modulating factors of cognitive efficiency are unspecific, can be difficult to control, and are not suitable to facilitate neural processing in a regionally specific manner. Here, we non-pharmacologically manipulated regionally specific brain activity using technically sophisticated real-time fMRI neurofeedback. This was accomplished by training participants to simultaneously control ongoing brain activity in circumscribed motor and memory-related brain areas, namely the supplementary motor area and the parahippocampal cortex. We found that learned voluntary control over these functionally distinct brain areas caused functionally specific behavioral effects, i.e. shortening of motor reaction times and specific interference with memory encoding. The neurofeedback approach goes beyond improving cognitive efficiency by unspecific psychological factors such as attention, arousal, or motivation. It allows for directly manipulating sustained activity of task-relevant brain regions in order to yield specific behavioral or cognitive effects.

## Highlights

• Neurofeedback training of motor cortex shortens reaction times.
• Self-regulation of parahippocampal cortex activity interferes with memory encoding.
• Differential neurofeedback reveals double dissociation between neurofeedback target areas.

## 1 Introduction

Perception, memory, and performing a motor task depend on specific patterns of brain activity. These patterns of brain activity can be divided into transient activity elicited by the stimuli or events, and sustained activity that precedes the stimuli/events. Recent evidence indicates that both pre- and post-stimulus activity contribute to task performance (Arieli, Sterkin, Grinvald, & Aertsen, 1996; Boly et al., 2007; Fox & Raichle, 2007; Fox, Snyder, Vincent, & Raichle, 2007; Hesselmann, Kell, Eger, & Kleinschmidt, 2008a; Hesselmann, Kell, & Kleinschmidt, 2008b; Ress, Backus, & Heeger, 2000). While the latter is largely determined by the stimulus characteristics itself, the former can be modulated by attention, arousal, and motivation (Broadbent, 1971; Freeman, 1933; James, 1890; Wundt, 1882). Although such modulating factors play an important role in task performance, they are rather general factors of cognitive efficiency that cannot facilitate regionally specific brain processes.

Here, we manipulated sustained brain activity in circumscribed brain regions by using real-time functional magnetic resonance imaging (fMRI) based neurofeedback. Rather than modulating sustained pre-stimulus activity in an unspecific way (e.g. via arousal), this new approach allowed us to train participants to voluntarily ‘clamp’ pre-stimulus levels of regionally specific brain activity at high or low levels. Until now, neurofeedback was mainly used to train self-regulation of autonomic functions or of specific electroencephalography (EEG) components, in order to communicate with severely paralyzed patients (Birbaumer et al., 1999; Birbaumer, Murguialday, & Cohen, 2008; Kübler, Kotchoubey, Kaiser, Wolpaw, & Birbaumer, 2001), to suppress epileptic activity (Kotchoubey et al., 2001; Sterman & Egner, 2006; Tan et al., 2009), or to treat symptoms of attention deficit hyperactivity disorder (Fuchs, Birbaumer, Lutzenberger, Gruzelier, & Kaiser, 2003; Gevensleben, Rothenberger, Moll, & Heinrich, 2012; Moriyama et al., 2012). However, neurofeedback with EEG is limited with respect to spatial specificity, and thus of the brain regions which can be targeted. Neurofeedback with real-time fMRI offers the advantage of learning to control spatially localized brain activity within the range of millimeters (Birbaumer, Ruiz, & Sitaram, 2013; deCharms, 2007, 2008; Sulzer et al., 2013a; Weiskopf et al., 2004; Weiskopf et al., 2007). So far, few studies have employed this technically challenging method, however, the existing ones have demonstrated the feasibility of self-regulating activation in specific brain areas. Some studies have additionally shown that self-regulation leads to behavioral effects that are specific to the functional role of the targeted cortical area (Bray, Shimojo, & O’Doherty, 2007; Caria et al., 2007; deCharms et al., 2005; Rota et al., 2009; Scharnowski, Hutton, Josephs, Weiskopf, & Rees, 2012; Shibata, Watanabe, Sasaki, & Kawato, 2011; Weiskopf et al., 2003, 2004). Recently, studies have even demonstrated therapeutic effects of real-time fMRI neurofeedback training in chronic pain patients (deCharms et al., 2005), Parkinson's disease (Subramanian et al., 2011), tinnitus (Haller, Birbaumer, & Veit, 2010), and depression (Linden et al., 2012).

Most neurofeedback studies so far have trained participants to control activity within one region of interest (ROI). This was accomplished by either providing feedback from the ROI alone (Bray et al., 2007; Caria, Sitaram, Veit, Begliomini, & Birbaumer, 2010; Johnson et al., 2012; Johnston et al., 2011; Johnston, Boehm, Healy, Goebel, & Linden, 2010; Koush, Zvyagintsev, Dyck, Mathiak, & Mathiak, 2012; Mathiak et al., 2010; Subramanian et al., 2011; Weiskopf et al., 2003; Yoo et al., 2007; Yoo, Lee, O’Leary, Panych, & Jolesz, 2008), or by providing differential feedback between the ROI and either the contralateral homologue of the ROI (Chiew, LaConte, & Graham, 2012; Robineau et al., 2014) or some kind of background region (e.g. a reference slice) (Caria et al., 2007; deCharms et al., 2004; deCharms et al., 2005; Haller et al., 2010; Hamilton, Glover, Hsu, Johnson, & Gotlib, 2011; Hampson et al., 2011; Rota et al., 2009; Veit et al., 2012). Differential feedback has the advantage that global effects such as breathing, heart rate, unspecific changes due to arousal, and head movements are less likely to cause artifactual self-regulation. This is because these sources of artifacts affect the ROI as well as the background region, and are canceled out with differential feedback. In the present study, we extended the use of differential feedback by now using a second, functionally unrelated ROI instead of an unspecific background region, and by also including bidirectional control of the feedback signal (participants learned to voluntarily up- and down-regulate the feedback signal). Such bidirectional control also excludes that self-regulation can arise from unspecific effects related to task demands, such as attention or arousal. Any unspecific effects that are related to task demands will only allow to either increase or decrease the differential feedback signal, but will not allow bidirectional control.

**Fig. 1:**
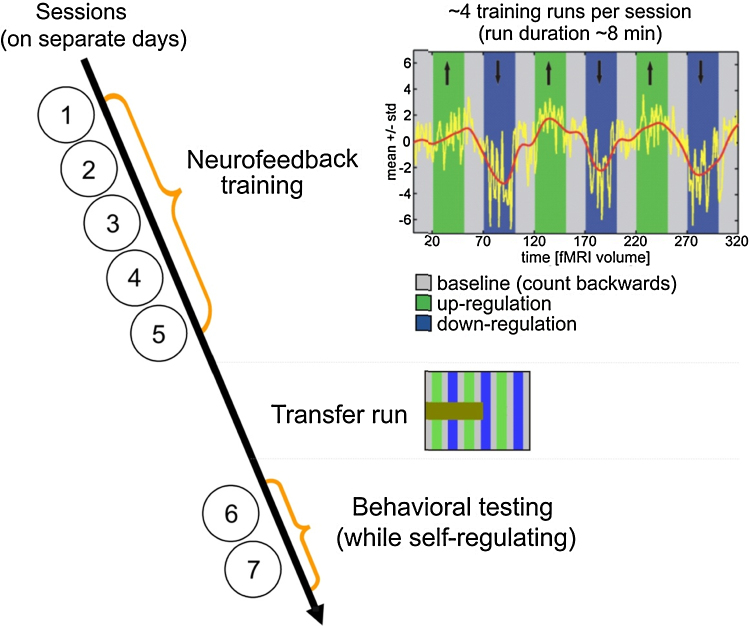
*Experimental design.* In order to learn simultaneous control over the level of ongoing activity in the SMA and in the PHC, participants underwent 12–22 runs of neurofeedback training spread over the course of 4-6 days, until they reached a pre-defined threshold of successful self-regulation. Each scanning session lasted ∼1 h. At the beginning of each neurofeedback training session, the ROIs were defined with functional localizers. Then, participants did on average 4 feedback runs of 8 min each per session. A feedback run was composed of 30 s baseline blocks (gray) interleaved with 45 s up- (green) and down-regulation (blue) blocks. The differential feedback signal was presented as a continuously updated yellow curve which was superimposed on the color-coded background illustrating the paradigm. For illustration purposes, a low-pass filtered (Gaussian FWHM = 25) version of the feedback signal is shown in red (this red curve and the black arrows were not presented during the experiment). After the training, participants tried self-regulation in the absence of feedback (transfer run), i.e. only the condition was indicated by a progress bar but not the feedback signal. Last, behavioral testing was performed in two separate scanning sessions on two separate days. (For interpretation of the references to color in this figure legend, the reader is referred to the web version of this article.)

The ROIs we trained were the supplementary motor area (SMA), which is involved in the control of movement (Grefkes, Eickhoff, Nowak, Dafotakis, & Fink, 2008; Koeneke, Lutz, Wustenberg, & Jancke, 2004; Nachev, Kennard, & Husain, 2008; Tanji, 2001), and the parahippocampal cortex (PHC), which is involved in memory encoding of visual scenes (Brewer, Zhao, Desmond, Glover, & Gabrieli, 1998; Gabrieli, Brewer, Desmond, & Glover, 1997; Stern et al., 1996; Turk-Browne, Yi, & Chun, 2006) and words (Fernandez, Brewer, Zhao, Glover, & Gabrieli, 1999; Otten, Quayle, Akram, Ditewig, & Rugg, 2006; Wagner et al., 1998). Because these two ROIs serve different brain functions, our paradigm involves the simultaneous training of two functionally distinct brain areas. We hypothesize that simultaneous differential training of the SMA and the PHC will cause behavioral effects that are linked to the functional role of each trained ROI. Specifically, we hypothesized that higher levels of SMA activity cause faster motor reaction times, and that higher levels of PHC activity cause improved memory. To test this hypothesis, we examined whether exercising voluntary control over SMA and PHC after neurofeedback training caused specific performance changes in a motor reaction time task and in a word memory task, respectively (Fig. 1).

## 2 Materials and methods

### 2.1 Functional MRI data acquisition

All experiments were performed on a 3 T Magnetom Trio scanner, using a standard transmit-receive head coil (Siemens Healthcare, Erlangen, Germany). Functional data were acquired with a single-shot gradient echo planar imaging sequence (matrix size: 64 × 64; resolution: 3.3 × 3.3 × 5 mm; 16 oblique transversal-coronal slices; slice thickness: 6 mm; slice gap: 1 mm; echo time TE: 35 ms; repetition time TR: 1500 ms; flip angle: 70°; receiver bandwidth: 2000 Hz/Px). For offline superposition of functional activations over anatomical details, we collected from each participant a high resolution T1-weighted structural scan of the whole brain (3D MDEFT; 1 mm isotropic resolution; matrix size: 256 × 240 mm; field of view: 256 × 240 mm; 176 sagittal partitions; echo time TE: 2.4 ms; repetition time TR: 7.92 ms; inversion time: 910 ms; flip angle: 15°; readout bandwidth: 195 Hz/pixel; spin tagging in the neck with flip angle 160° in order to avoid flow artifacts) (Deichmann et al., 2004).

The neurofeedback setup used Turbo-BrainVoyager (Brain Innovation, Maastricht, The Netherlands), custom real-time image export tools programmed in ICE VA25 (Siemens Healthcare) (Weiskopf et al., 2004), and custom scripts running on MATLAB (Mathworks Inc., Natick, MA, USA). The setup allowed participants to observe BOLD signal changes in specific brain regions with a delay of less than 1.5 s from the acquisition of the image. Head motion was corrected in real-time using Turbo-BrainVoyager.

### 2.2 Participants

Seven naïve adult volunteers (1 male, ages between 23 and 26 years, all right handed) with normal or corrected-to normal vision gave written informed consent to participate in the experiment. The experimental protocol was approved by the local ethics committee of the Faculty of Medicine, University of Tübingen, Germany. Before the experiment, they completed standardized tests assessing their spatial orientation ability (Stumpf & Fay, 1983), creative imagination (Barber & Wilson, 1978), and mood (Zerssen, 1976).

Participants received written instructions describing that they will be able to see their brain activity during the scanning and that they should raise or lower the feedback signal in accordance with the paradigm. The instructions included an explanation of the neurofeedback display (Fig. 1), and an explanation that they should not move and that they should breathe regularly. We also explained to the participants that the feedback was delayed by approximately 8 s (the hemodynamic delay plus the real-time analysis processing time). No background information about the differential feedback signal, or the anatomical areas was given. Initially, we also did not recommend any cognitive strategies for controlling the feedback signal. In order to facilitate learning, 6 out of the 7 participants received instructions that imagery of movements and spatial navigation might help to modulate the feedback curve after the third neurofeedback training session. Explicit strategies like imagery of fist clenching, skiing, navigating, and views of buildings or places were suggested as potential regulation strategies. Nevertheless, it was emphasized that participants should find an individual strategy that worked best for them.

After each scanning session, participants were asked to complete a written questionnaire and amongst other questions, describe how they tried to manipulate the feedback signal, how effective their strategy was, and how they rated the attentional demands.

### 2.3 Functional localizer runs

**Fig. 2:**
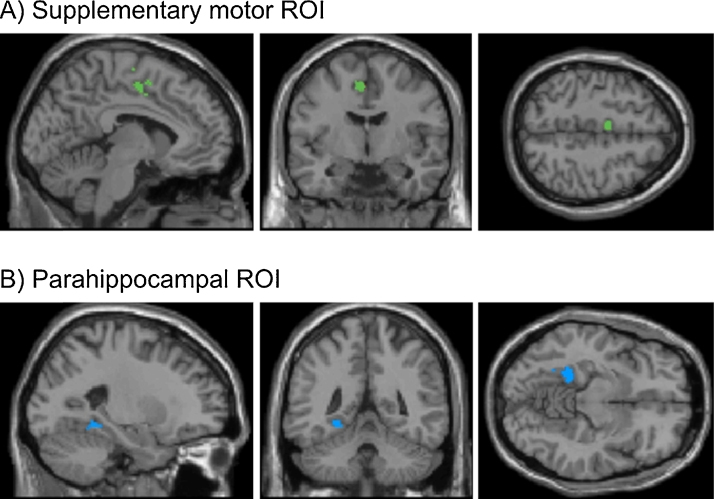
*Illustration of the (A) SMA (green) and the (B) PHC ROIs (blue).* In order to generate the average group ROIs, we first determined each participant's ROI based on all localizer runs, and then averaged over all participants. During the neurofeedback runs, the SMA ROI enclosed on average 26 voxels, and the PHC ROI 22 voxels. (For interpretation of the references to color in this figure legend, the reader is referred to the web version of this article.)

Each neurofeedback training session began with two functional localizer runs to delineate the ROIs from which the participants received feedback (Fig. 2). The first localizer run was used to define the SMA ROI. It consisted of five baseline blocks separated by three blocks of bimanual finger tapping. The second localizer run was used to define the PHC ROI. It consisted of three blocks of presentation of outdoor scenes alternating with three blocks of presentation of faces. All of these blocks were separated by baseline blocks. Block length was always 45 s. During baseline blocks, participants were instructed to count down from 100. Visual stimuli and instructions during localizer and feedback runs were displayed using a large circular projection screen at the rear of the scanner bore with a mirror positioned within the head-coil.

The SMA ROI for neurofeedback was restricted to those voxels in a rectangular region anterior of the paracentral sulcus and superior of the cingulate sulcus that exhibited a positive BOLD response to the finger tapping (*p* < 0.01, Bonferroni corrected for multiple comparisons). The PHC ROI for neurofeedback was restricted to those voxels in a rectangular region around the left parahippocampal gyrus that exhibited a greater BOLD response to houses in contrast to faces (*p* < 0.01, Bonferroni corrected for multiple comparisons).

### 2.4 Neurofeedback training

For each training session, participants performed on average 4 training runs of 8 min each. The training runs were composed of three 45 s up-regulation blocks and three 45 s down-regulation blocks, which were all interleaved with 30 s baseline blocks (Fig. 1). The blocks were color coded to indicate up-/or down-regulation blocks. During up-regulation blocks, the participants should increase the feedback signal. During down-regulation blocks, the participants should decrease the feedback curve. During baseline blocks, participants were instructed to mentally count backwards from 100. The order of up- and down-regulation blocks and the color assignment to up- and down-regulation was pseudo-randomized between participants, i.e. three volunteers were trained to up-regulate in regulation blocks 1,3, and 5, whereas four volunteers up-regulated in regulation blocks 2,4, and 6. Also the type of feedback signal was pseudo-randomized: Three volunteers were trained to control the SMA-PHC feedback signal, and four volunteers were trained to control the PHC-SMA feedback signal.

Participants were presented feedback via a continuously updated yellow curve which was superimposed on the color-coded background illustrating the condition (i.e. baseline, up-regulation, or down-regulation). The yellow curve represented the difference between the BOLD response of the SMA ROI and the PHC ROI, i.e. SMA-PHC for some and PHC-SMA for other participants. The differential feedback signal was normalized in relation to the mean and the standard deviation of the first baseline block. The maximum amplitude of the display was set to 10 standard deviations of the first baseline block.

After each run, self-regulation performance was quantified in SPM99 using a GLM consisting of 2 regressors indicating up- and down-regulation. Motion parameters (translation, rotation) were included as covariates to reduce the impact of residual motion artifacts. This GLM was not applied to the whole brain but only to the high-pass filtered (5.55 × 10^−3^ Hz) differential feedback signal. Both regressors were contrasted to determine signal differences between up- and down-regulation and the corresponding t-values were calculated. This analysis was carried out only to determine the further course of the experiment, and was not presented to the participants. Also, for the offline analysis, different statistical procedures were used. The neurofeedback training procedure was repeated until participants achieved a pre-defined criterion of successful self-regulation, i.e. when a t-value higher than 3.1 (which is equivalent to *p* < 0.001) was reached. Across participants, the training objectives were reached within 12–22 runs spread over the course of 4–6 days.

After successful neurofeedback training, participants performed self-regulation in the absence of feedback (transfer run). For this, the feedback curve was replaced by a large brown bar which provided only information about the timeline and the condition (i.e. baseline, up-regulation, or down-regulation) but not about the brain activations.

### 2.5 Behavioral test runs

A few days after the neurofeedback training, behavioral testing during self-regulation was performed in two separate scanning sessions spread over the course of two days, i.e. they were completely independent from the neurofeedback training and transfer sessions. During the behavioral test runs, the participants did not receive neurofeedback information.

For the reaction time test, participants had to perform one of two acoustically triggered bimanual finger sequences while self-regulating. A high (3000 Hz) or low pitch tone (1500 Hz) applied via headphones indicated the type of finger sequence. Both tones were presented for 100 ms at ∼80 dB Sound Pressure Level (SPL). When the high pitch tone was presented, participants had to press buttons of a MR compatible response box with their left index finger (D2), their right D2, their left middle finger (D3), and their right D3. For the low pitch tone the sequence of button presses was right D3, left D3, right D2, and left D2. In order to avoid anticipatory responses, the inter-stimulus interval of the acoustic cues was pseudo-randomized from 6 to 15 s. On average, the acoustic cues were presented every 10.5 s. During four test runs (same configuration as for the transfer runs), a total of 72 high and low pitch tones were presented during up- and down-regulation blocks. Motor sequences were recorded in real time with custom-made software (Muster5, MEG-Center, University of Tübingen, Germany). Before the test runs, subjects underwent 300 trials of pre-training outside of the scanner to become acquainted with the task. Reaction times were corrected for outliers by removing trials that were more than 2 standard deviations away from the mean. Only correct trials were analyzed. In order to assess reaction time differences between the up- and down-regulation blocks, a paired *t*-test was applied on the group level (two-tailed; statistical significance threshold of *p* < 0.05).

For the word memory task, participants had to process words while self-regulating. Due to technical problems, only six out of the seven participants performed this task. These words were presented in capital letters every six seconds above the feedback display. They were presented for 1.5 s and encompassed approximately 5° of visual angle. During baseline blocks, no words were presented. To ensure that the participants attended to the words, they were asked to detect and indicate randomly interspersed pseudo words by pressing a button. Participants were not instructed to memorize the words, and they did not know that their memory for these words was later on assessed by an unexpected word recognition test. During 3 test runs (same configuration as for the transfer runs), a total of 123 words (3 × 35 words and 3 × 6 pseudo words) were presented. They were nouns or verbs out of three semantic categories: space, movement, and neutral (consisting of words related to animals or food). Between categories, all words were balanced for frequency, type, category, and length according to the Mannheim Corpus of German language (Institut fuer Deutsche Sprache, Mannheim, Germany).

Approximately 20 min after the last imaging run, participants were administered an unexpected recognition test. For this, they were presented with a written list of words, containing all the presented words and the same number of new words (which were semantically and linguistically balanced as the test words). Participants had to indicate if a given word had been presented during any of the 3 preceding behavioral test scans by tagging ‘sicher’ (German for ‘sure’) if they were absolutely sure of having seen the word, ‘unsicher’ (German for ‘unsure’) in case it appeared familiar, and ‘neu’ (German for ‘new’) otherwise. A word that has been presented during the behavioral test scans was classified as remembered when the participant was absolutely sure or believed to have seen the word during the scanning. A word that has been presented during the recognition test scans was classified as forgotten when the participant labeled it as new. In order to assess word memory differences between the up- and down-regulation blocks, recognition performance was compared on the group level using a sign rank test (statistical significance threshold of *p* < 0.05).

### 2.6 Offline analysis

#### 2.6.1 Initial offline data preprocessing

Offline data analysis was performed using SPM8 (Wellcome Trust Centre for Neuroimaging, Queen Square, London, UK; http://www.fil.ion.ucl.ac.uk/) and BrainVoyager QX (Brain Innovation). The first 10 volumes of each run were excluded from statistical analysis to allow for T1-related equilibration. The remaining images were corrected for slice time acquisition differences, realigned to the first scan of each run, coregistered to the structural scan, normalized to MNI space, and smoothed with an isotropic Gaussian kernel with 4 mm full-width-at-half-maximum (FWHM).

#### 2.6.2 Offline ROI analysis

In order to assess the neurofeedback training success, we specified GLMs with regressors for the optimal differential feedback signal, the optimal SMA time course, or the optimal PHC time course. For example, if the participant was trained to up-regulate the differential feedback signal SMA-PHC in regulation blocks 2, 4, and 6 (and consequently to down-regulate in regulation blocks 1, 3, and 5), then the regressors for the optimal differential feedback signal as well as the optimal SMA time course was set to 1 in regulation blocks 2, 4, and 6, and to −1 in regulation blocks 1, 3, and 5. The optimal PHC time course regressor in this example was set to –1 in regulation blocks 2, 4, and 6, and to 1 in regulation blocks 1, 3, and 5. The regressors were modeled as boxcar functions convolved with the canonical hemodynamic response function (HRF) in SPM8. The beta parameter estimates were computed separately for each ROI time course of each run. Per GLM, only a single regressor was used, i.e. the optimal differential feedback signal regressor for the differential feedback time course, the optimal SMA time course regressor for the SMA time course, and the optimal PHC time course regressor for the PHC time course.

Because the number of completed training runs varied slightly across participants, i.e. participants completed 19, 20, 17, 17, 12, 19, or 22 training runs, the beta parameter estimates for each participant were grouped and averaged into 12 bins (=minimum number of training runs that all participants completed) in a nearest neighbor fashion. To assess the neurofeedback learning effect, linear regressions of the mean beta parameter estimates over training runs were calculated for the differential feedback signal, for the SMA time course, and for the PHC time course. In addition, *t*-tests were calculated to examine regulation success in the training runs, in the transfer runs, and in the behavioral test runs (two-tailed; statistical significance threshold of *p* < 0.05). The same analyses were carried out separately for participants that were trained to control SMA-PHC and for participants that were trained to control PHC-SMA.

In order to investigate the relative contribution of the SMA and the PHC to up- vs. down-regulation of the differential feedback signal, we separately computed the percentage of signal change in the SMA and the PHC of the last 5 training runs (these runs showed the best control over the feedback signal). This was done separately for SMA_up_/PHC_down_ blocks, and for SMA_down_/PHC_up_ blocks. The time courses were normalized so that the percentage of signal change during baseline activity corresponded to 0%, i.e. the average baseline signal change was subtracted from each time point during the regulation blocks.

#### 2.6.3 Whole brain analyses

In first level analysis, we specified GLMs with regressors for the up-regulation condition, and covariates derived from head movement parameters to capture residual motion artifacts. The regressors were modeled as boxcar functions convolved with the canonical hemodynamic response function (HRF) in SPM8. In second level, we calculated fixed-effect group analyses contrasting self-regulation vs. baseline, SMA_up_/PHC_down_ blocks vs. baseline, SMA_down_/PHC_up_ blocks vs. baseline, and SMA_up_/PHC_down_ blocks vs. SMA_down_/PHC_up_ blocks of the last training run. For the comparisons with baseline, a positive contrast was applied to reveal brain activations, and a negative contrast to reveal deactivations. Statistical parametric maps were thresholded at *p* < 0.05 corrected for multiple comparisons using the family wise error rate (FWE). The averaged ROIs were used to perform small volume correction of the whole brain analyses (Worsley et al., 1996). Random-effects analyses did not reveal significant effects due to the low number of participants (except for significant changes in the ROIs after small volume correction based on the average ROI). The results of the whole brain analyses therefore cannot be generalized beyond the study sample.

#### 2.6.4 Exploration of connectivity changes using psychophysiological interaction (PPI) analysis

To explore connectivity changes due to learned self-regulation, we conducted a psychophysiological interactions analysis (PPI, Friston et al., 1997) between different brain areas and activity in the ROIs. For the PPI analysis, we specified general linear models (GLMs) with regressors for the respective ROI time course, for the experimental conditions (i.e. a boxcar function representing up- and down-regulation and baseline blocks convolved with the canonical hemodynamic response function in SPM8), and for the interaction between the two. This was done separately for the SMA ROI and the PHC ROI time courses. To reveal areas of the brain whose connectivity to the respective ROI changed depending on self-regulation, we first applied a positive contrast to parameters estimated for the interaction term, and then calculated a voxelwise 1-sample *t*-test of the interaction term contrast images of each participant's last training run. Statistical parametric maps were thresholded at *p* < 0.05 corrected for multiple comparisons using FWE.

In addition to exploring psychophysiological interactions across the whole brain, we specifically assessed the interaction between the SMA ROI with the PHC ROI. For this, we extracted the mean PPI parameter estimate at the location overlapping with the SMA ROI from the whole brain PPI analysis that was based on the PHC ROI time course. Vice versa, to reveal the interaction between the PHC ROI with the SMA ROI, we extracted the mean PPI parameter estimate at the PHC ROI location from the whole brain PPI analysis that was based on the SMA ROI time course. This was done for each participant and for each neurofeedback training run. In order to assess interaction changes across training runs, we calculated a linear regression of the group average over training runs (statistical significance threshold of *p* < 0.05).

#### 2.6.5 Correlation between reaction times and ROI activity

To investigate how voluntary self-regulation influenced reaction times, we correlated the activity in the ROIs at the time of the acoustic motor response trigger with reaction times of the respective trial (i.e. the time it took until the finger sequence was initiated). For this, we calculated the mean percentage of signal change in the regulation blocks compared to the baseline blocks for each run, and we z-transformed the reaction times to allow for between-subject comparison. Finally, we performed a linear regression analysis correlating the activity at the SMA ROI as well as the PHC ROI at the time of the acoustic cue and the reaction times. The factor subjects were included as a random effects variable of no interest to account for inter-subject variance. The same correlation analysis was carried out for the duration of the finger tapping sequence, i.e. for the time it took to perform the complete finger tapping sequence.

In addition, we plotted the percentage of signal change time courses of the ROIs from the time of the auditory cue to the execution of the motor response. This was done separately for up- and down-regulation blocks. To compare each ROI's activity in the up-regulation vs. the down-regulation blocks at the time of the auditory cue, we calculated paired *t*-tests (two-tailed; statistical significance threshold of *p* < 0.05).

## 3 Results

### 3.1 Learning voluntary control of SMA and PHC

**Fig. 3:**
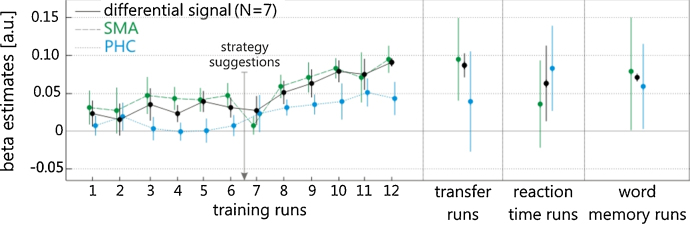
*Neurofeedback learning performance.* Self-regulation performance was measured as beta parameter estimates, which indicate how close the measured signal was following the regulation task. The 7 participants showed an increase in differential feedback signal control with training. This increase is mediated by voluntarily controlling both components of the differential feedback signal, i.e. the SMA and the PHC ROIs. Voluntary control was maintained during transfer runs and during the behavioral test runs. After the ∼6th training run, specific regulation strategies related to motor imagery and spatial navigation were suggested to the participants. Error bars represent one standard error of the mean. (For interpretation of the references to color in this figure legend, the reader is referred to the web version of this article.)

Each participant completed at least 12 neurofeedback training runs spread over the course of 4–6 days. Over the course of this training, participants successfully learned to control the differential feedback signal. Specifically, participants showed a significant increase in beta parameter estimates for the differential feedback signal associated with training (Fig. 3; differential signal linear regression: *r*^2^ = 0.83, *F*(1,10) = 47.39, *p* < 0.01). An exemplary time course of successful regulation of the differential feedback signal is shown in Fig. 1. Participants accomplished this by learning to self-regulate both the SMA (Fig. 3; SMA linear regression: *r*^2^ = 0.56, *F*(1,10) = 12.58, *p* < 0.01) as well as the PHC (Fig. 3; PHC linear regression: *r*^2^ = 0.71, *F*(1,10) = 23.87, *p* < 0.01) components of the differential feedback signal.

This learned ability to control the differential feedback signal was subsequently maintained in the absence of neurofeedback. This was shown in transfer runs, where we tested the ability of trained participants to regulate the differential feedback signal in accordance with the paradigm, but this time in the absence of neurofeedback (Fig. 3, transfer run column; differential signal 1-sample *t*-test: *t*(6) = 8.41, *p* < 0.01; see Fig. 1 for an illustration of the experimental display during transfer runs). During the transfer runs, regulation of the differential feedback signal was accomplished by mainly controlling activity in the SMA (Fig. 3, transfer run column; SMA 1-sample *t*-test: *t*(6) = 4.27, *p* < 0.01; PHC 1-sample *t*-test: *t*(6) = 1.36, *p* = 0.22).

Because we pseudo-randomized the type of feedback signal (i.e. three volunteers were trained to control the SMA-PHC feedback signal, and four volunteers were trained to control the PHC-SMA feedback signal), we also investigated performance separately for these respective sub-groups. Learning control of the differential feedback signal did not depend on whether the participants received SMA–PHC or PHC–SMA feedback (see Supplemental Fig. S1).

**Fig. 4:**
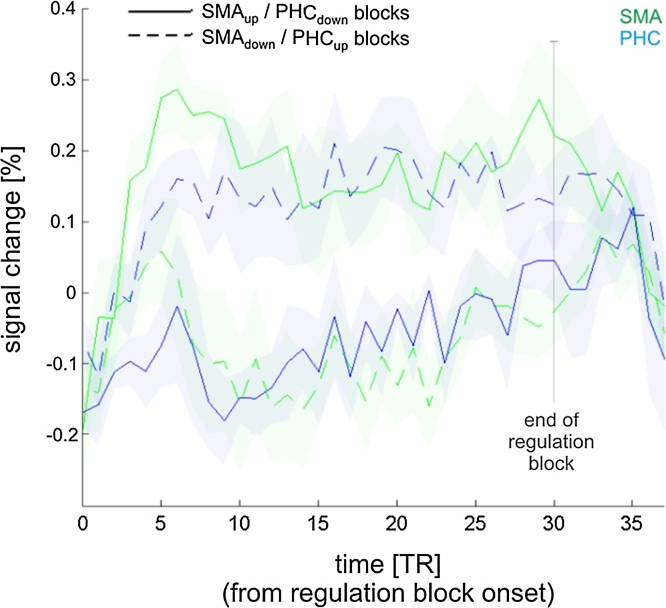
*ROI time courses separately for SMA_up_/PHC_down_ and SMA_down_/PHC_up_ blocks of the last training runs.* SMA activity (green) was increased during SMA_up_/PHC_down_ blocks (solid lines), and decreased during SMA_down_/PHC_up_ blocks (dashed lines). In contrast, PHC activity (blue) was decreased during SMA_up_/PHC_down_ blocks, and increased during SMA_down_/PHC_up_ blocks. This illustrates that successful self-regulation was accomplished by up- and down-regulating the respective ROI. Shaded areas represent one standard error of the mean. The time courses were normalized so that the percentage of signal change during baseline activity corresponded to 0%.(For interpretation of the references to color in this figure legend, the reader is referred to the web version of this article.)

Both up- and down-regulation of each ROI contributed to successful self-regulation. During SMA_up_/PHC_down_ blocks, activity in the SMA was increased and at the same time activity in the PHC was decreased (Fig. 4). Likewise, during SMA_down_/PHC_up_ blocks, activity in the SMA was decreased and activity in the PHC was increased. Please note that the distinction into SMA_up_/PHC_down_ and SMA_down_/PHC_up_ blocks was done post-hoc, i.e. the participants were not aware of this distinction and were only instructed to up- and down-regulate the feedback signal (see Section 2 for details).

### 3.2 Neural processes underlying control of the differential feedback signal

**Fig. 5:**
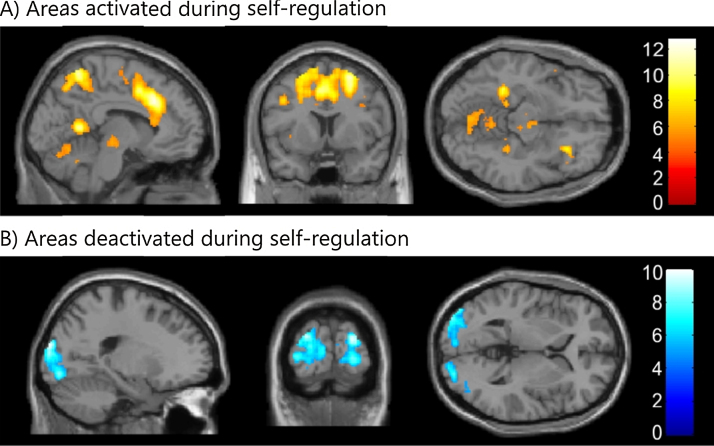
*Whole brain analyses.* Shown are brain activation maps for (A) increased activity during self-regulation blocks, and (B) deactivations during self-regulation blocks. Activation increases during self-regulation blocks included the SMA and the PHC ROIs, the middle cingulate cortex bilaterally, the left superior parietal lobe, the right superior frontal gyrus, the precuneus bilaterally, the cerebellum bilaterally, the inferior parietal cortex bilaterally, the right hippocampus, and the left putamen. Activation decreased in the superior and ventral visual cortex bilaterally. The figures show contrast maps thresholded at *p* < 0.05 (corrected for multiple comparison using FWE) on the MNI template brain. For details, see Table 1.

Whole-brain group-level analyses revealed specific patterns of brain activity during self-regulation blocks, i.e. SMA_up_/PHC_down_ and SMA_down_/PHC_up_ blocks combined. Activation increases during self-regulation blocks included the SMA and the PHC ROIs, the middle cingulate cortex bilaterally, the left superior parietal lobe, the right superior frontal gyrus, the precuneus bilaterally, the cerebellum bilaterally, the inferior parietal cortex bilaterally, the right hippocampus, and the left putamen (Fig. 5A; Table 1). Activation decreased in the superior and ventral visual cortex bilaterally (Fig. 5B; Table 1).

**Fig. 6:**
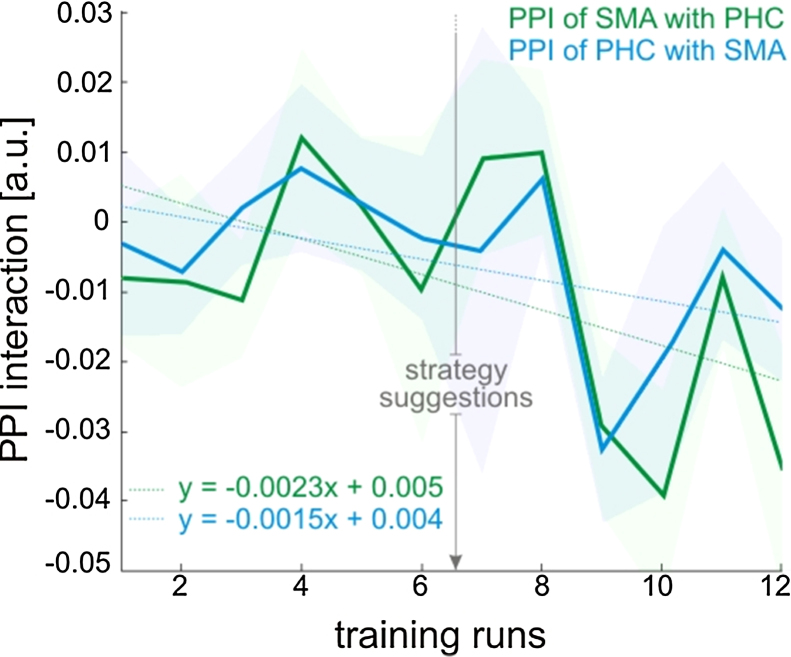
*Psychophysical interaction between the SMA and the PHC across neurofeedback training.* With training, there was a trend towards an increase in negative coupling between the SMA and the PHC across the training sessions (Fig. 6; PPI of SMA with PHC linear regression: *r*^2^ = 0.217, *F*(1,10) = 2.77, *p* = 0.13; PPI of PHC with SMA linear regression: *r*^2^ = 0.232, *F*(1,10) = 3.01, *p* = 0.11). Shaded areas represent one standard error of the mean.

To provide further insight into the neural processes underlying control of the differential feedback signal, we explored psychophysiological interactions (PPI) with activity in the SMA ROI as well as the PHC ROI (Friston et al., 1997). The explorative whole brain PPI analysis revealed no region whose connectivity to the SMA or the PHC changed significantly. However, when inspecting the ROIs themselves, we found that during self-regulation, activity in the SMA ROI was negatively coupled to activity in the PHC ROI (Fig. 6; PPI of SMA with PHC 1-sample *t*-test of the last training run: *t*(6) = −2.47, *p* = 0.04). Naturally, activity in the PHC ROI was also negatively coupled to activity in the SMA ROI, although this coupling did not reach significance (Fig. 6; PPI of PHC with SMA 1-sample *t*-test of the last training run: *t*(6) = −1.13, *p* = 0.30). There was a trend towards an increase in negative coupling between the SMA and the PHC across the training sessions (Fig. 6; PPI of SMA with PHC linear regression: *r*^2^ = 0.217, *F*(1,10) = 2.77, *p* = 0.13; PPI of PHC with SMA linear regression: *r*^2^ = 0.232, *F*(1,10) = 3.01, *p* = 0.11).

### 3.3 Cognitive processes underlying control of the differential feedback signal

As part of the debriefing after the neurofeedback training sessions, participants were asked how they attempted to regulate the feedback signal. Initially, the participants described their imagery as, for example, ‘having positive (for up-regulation) or negative (for down-regulation) emotions’, ‘thinking of flying birds (for up-regulation) or diving fish (for down-regulation), or they tried to control the feedback by looking at the location where they wanted the feedback signal to be. However, these cognitive strategies did not work for all but one participant, and successful self-regulation was achieved only after we recommended potential regulation strategies that were related to the functional role of the SMA and PHC (Fig. 3; strategy suggestions; see Section 2 for details). For all but one participant, after the second training day, we suggested the use of motor and spatial navigation imagery, but we did not specify when these regulation strategies might be most effective. Following our recommendations, some participants increased activity in the SMA by, for example, imagining ‘playing the piano’, ‘doing sports’, or ‘dancing’. To increase activity in the PHC, some participants described their imagery as ‘navigating home’, ‘driving home’, or ‘walking through the apartment’.

However, we emphasized that participants had to find their own best strategy, and some of their cognitive strategies did not fit into the category of motor-related or navigation-related imagery. For example, one participant, who received SMA-PHC feedback, ‘imagined numbers’ in order to up-regulate the feedback signal and ‘relaxed’ or ‘imagined dark colors’ in order to down-regulate the feedback signal. This participant nevertheless successfully learned to control the differential feedback signal (differential feedback signal 1-sample *t*-test across all training runs: *t*(19) = 3.36, *p* < 0.01). For this participant, the PHC ROI contributed more to successful self-regulation (PHC 1-sample *t*-test across all training runs: *t*(19) = 2.92, *p* < 0.01) than did the SMA ROI (SMA 1-sample *t*-test across all training runs: *t*(19) = 1.79, *p* < 0.08).

One participant even successfully controlled the differential feedback signal without receiving recommendations for potential regulation strategies (differential feedback signal 1-sample *t*-test across all training runs: *t*(11) = 8.98, *p* < 0.01). This participant received PHC-SMA feedback and thought about ‘the effects of radiation therapy’ or ‘imagined the layout of an x-ray machine’ in order to increase the feedback signal. In order to decrease the feedback signal this participant ‘imagined their work environment/apartment’. Given the functional role of the PHC, especially the latter strategy should increase rather than decrease the PHC activity and consequently not lead to a decreasing differential feedback signal. Indeed, we found that this participant learned to regulate the differential feedback signal by regulating the SMA (SMA 1-sample *t*-test across all training runs: *t*(11) = 10.57, *p* < 0.01), but that she did not learn to regulate the PHC (PHC 1-sample *t*-test across all training runs: *t*(11) = 1.14, *p* = 0.28).

In order to elucidate the psychological underpinnings of successful self-regulation, we also assessed the participants’ ability in spatial orientation (Stumpf & Fay, 1983), creative imagination (Barber & Wilson, 1978), and mood (Zerssen, 1976). However, none of these psychological questionnaires was predictive with respect to regulation success (all *p*s > 0.05).

### 3.4 Behavioral effects of self-regulation: The reaction time task

**Fig. 7:**
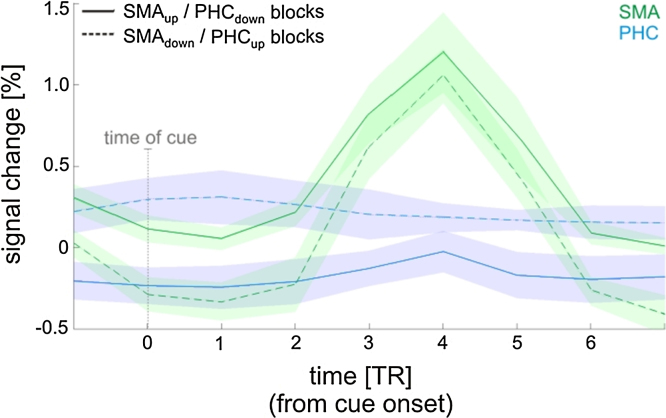
*ROI time courses at the time of the auditory cue presentation.* During the reaction time task session, participants achieved a significant increase in SMA activity (green) in SMA_up_/PHC_down_ blocks compared to SMA_down_/PHC_up_ blocks at the time of the cue. The opposite can be found for the PHC (blue), which indicates that both ROIs were successfully modulated during this session. Also, the effect of the overt motor response is visible as increasing SMA activity a few time points after the auditory cue. Shaded areas represent one standard error of the mean.

During the behavioral test session, participants showed significant control over the differential feedback signal (Fig. 3, SMA test run column; differential signal 1-sample *t*-test: *t*(6) = 2.97, *p* = 0.02). As anticipated, due to the simultaneous behavioral experiment, self-regulation of the differential feedback signal was somewhat less successful than during the training and transfer runs, but it still demonstrated statistically significant differences from baseline. Likewise, self-regulation of the SMA ROI alone was no longer evident over the behavioral test session because it was masked by SMA activity related to overt finger movements (Fig. 3, SMA test run column; SMA 1-sample *t*-test: *t*(6) = 1.60, *p* = 0.16). However, at the time of the auditory cue, which triggered the motor response, SMA activity was significantly higher during the SMA_up_/PHC_down_ blocks than during the SMA_down_/PHC_up_ blocks (Fig. 7; paired *t*-test: *t*(6) = 3.29, *p* = 0.02). The opposite pattern was found for the PHC (*t*-test: *t*(6) = –2.50, *p* = 0.04).

**Fig. 8:**
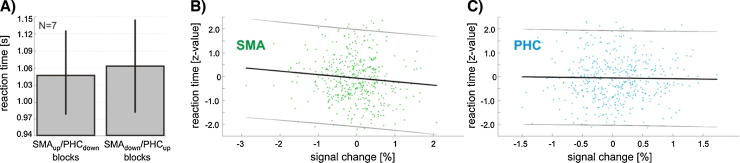
*Reaction times.* (A) There was no significant difference in reaction times between SMA_up_/PHC_down_ blocks compared to SMA_down_/PHC_up_ blocks. Error bars represent one standard error of the mean. (B) During the reaction time session, there was a significant negative correlation between self-regulated activity in the SMA at the time of the auditory cue presentation and reaction times. (C) Such a correlation was not found for the PHC. Please note the different scaling of the *x*-axes. Gray lines indicate 95% confidence intervals.

When collapsing across all trials, the increase in SMA activity (and the decrease in PHC activity) that the participants achieved during the test session was not associated with a significant decrease in reaction times (Fig. 8A paired *t*-test: *t*(6) = −0.56, *p* = 0.60).

There was, however, a significant negative correlation between the self-regulated activity in the SMA and the reaction times across trials over the behavioral test session, i.e. the more the participants increased/decreased activity in the SMA, the faster/slower they responded (Fig. 8B; SMA linear regression: *r*^2^ = −0.15, *F*(1,453) = 4.21, *p* = 0.04). Reaction times did not correlate with activity in the PHC ROI (Fig. 8C; PHC linear regression: *r*^2^ = −0.03, *F*(1,453) = 0.14, *p* = 0.71). This correlation was specific to the onset of the movement and was not evident for the duration of the movement, i.e. the time it took to perform the finger sequence (SMA linear regression: *r*^2^ = 0.04, *F*(1,453) = 0.29, *p* = 0.59; PHC linear regression: *r*^2^ = −0.04, *F*(1,453) = 0.23, *p* = 0.64).

### 3.5 Behavioral effects of self-regulation: The memory task

During the behavioral test session, participants showed significant control over the differential feedback signal (Fig. 3, PHC test run column; differential signal 1-sample *t*-test: *t*(6) = 3.97, *p* < 0.01). Again, due to the simultaneous behavioral experiment, self-regulation of the differential feedback signal was somewhat less successful than during the training and transfer runs, but it still demonstrated statistically significant differences from baseline. Self-regulation during the word memory task was evident in both the SMA ROI (Fig. 3, PHC test run column; SMA 1-sample *t*-test: *t*(6) = 2.75, *p* = 0.03) as well as in the PHC ROI (Fig. 3, PHC test run column; PHC 1-sample *t*-test: *t*(6) = 2.59, *p* = 0.04).

**Fig. 9:**
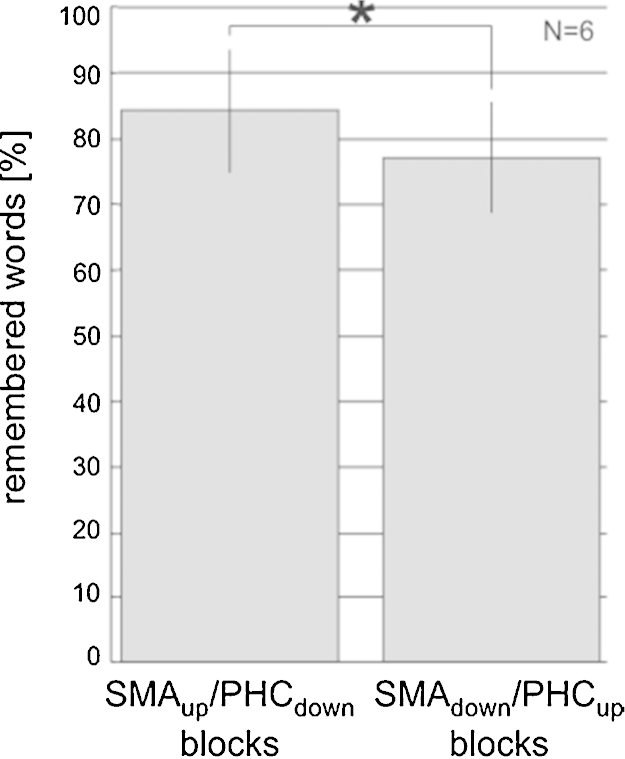
*Memory for words.* Participants remembered significantly more words that had been presented during SMA_up_/PHC_down_ blocks, compared to words that had been presented during SMA_down_/PHC_up_ blocks. Error bars represent one standard error of the mean.

The increase in PHC activity (or decrease in SMA activity) that the participants achieved during the test session was associated with a significant decrease in memory for words, and this in all participants (Fig. 9; Wilcoxon sign rank test: *p* = 0.03).

## 4 Discussion

Using differential real-time fMRI neurofeedback, we demonstrated that participants could learn to simultaneously control activity in the SMA and the PHC (Fig. 3). The control over the feedback signal was subsequently maintained in transfer runs where participants no longer received neurofeedback information. When participants voluntarily regulated activity in these regions, significant changes in motor reaction times and memory performance were observed, which were specific to the differential self-regulation.

### 4.1 Neurofeedback learning

Through neurofeedback training, the participants in our study achieved control over the differential feedback signal (Fig. 3). The control over the feedback signal was subsequently maintained in transfer runs where participants no longer received neurofeedback information, and in behavioral test runs where participants had to perform additional behavioral tests while self-regulating.

How was such control achieved by the participants? In order to elucidate the neural underpinnings of successful self-regulation, we analyzed how well the ROI activity changes followed the requirements of the experimental paradigm. We found that participants simultaneously regulated activity in the SMA as well as in the PHC, i.e. both ROIs contributed to successful self-regulation (Fig. 3; SMA in green, PHC in blue). However, this could have been achieved without true bidirectional control of each ROI, for example, by up-regulating the SMA during SMA_up_/PHC_down_ blocks (while the PHC remains unchanged) and up-regulating the PHC during SMA_down_/PHC_up_ blocks (while the SMA remains unchanged). We therefore analyzed the ROI time courses and found that the ROIs were not only up-regulated, but also down-regulated (Fig. 4). For example, when a participant received SMA-PHC differential feedback, then activity in the SMA increased during up-regulation blocks and decreased during down-regulation blocks, and activity in the PHC decreased during up-regulation blocks and increased during down-regulation blocks. This negative coupling between the SMA and the PHC was also evident in the PPI analysis, which revealed connectivity changes between both regions that changed with training (Fig. 6). Such bidirectional control excludes that self-regulation and the resulting behavioral consequences are due to unspecific effects such as attention or arousal, providing a strong within-subject control.

To shed further light on the neural substrate of neurofeedback learning, we applied whole brain analyses to reveal brain activations extending beyond the SMA and PHC ROIs. Similar to previous real-time fMRI neurofeedback studies, we found that self-regulation resulted in widespread brain activations (e.g. Chiew et al., 2012; Haller et al., 2013; Rota, Handjaras, Sitaram, Birbaumer, & Dogil, 2011; Subramanian et al., 2011; Sulzer et al., 2013b; Veit et al., 2012; Zotev et al., 2011). These activations included the SMA and PHC ROIs, attention-related parietal areas, cingulate areas which might be involved in reward-based learning, and areas related to skill learning such as the putamen (Fig. 5; Table 1). Especially the involvement of motor circuits and the basal ganglia are interesting, because they are consistent with a recently proposed theory according to which neurofeedback learning is akin to skill learning (Birbaumer et al., 2013). When considering only SMA_up_/PHC_down_ blocks, motor areas but not parahippocampal areas were activated (see Supplemental Fig. S2; Supplemental Table S1). Likewise, during SMA_down_/PHC_up_ blocks, the SMA was no longer activated (see Supplemental Fig. S2; Supplemental Table S1). This reflects the fact that up-regulation of the ROIs was more pronounced than their down-regulation (see also Fig. 3). Nevertheless, at a lower statistical threshold, brain activation maps also indicate deactivations of the respective ROI, i.e. of the PHC during SMA_up_/PHC_down_ blocks and of the SMA during SMA_down_/PHC_up_ blocks (not shown). Surprisingly, we found consistent deactivations in the visual cortex even though there was no difference in the visual display between regulation and baseline blocks, and despite the use of imagery as a cognitive control strategy during regulation blocks (Guillot et al., 2009; Kosslyn, Ganis, & Thompson, 2001; Slotnick, Thompson, & Kosslyn, 2005; Stokes, Thompson, Cusack, & Duncan, 2009).

In order to elucidate the cognitive processes underlying successful self-regulation, we debriefed the participants after the neurofeedback training sessions. As part of the debriefing, participants described the contents of their imagery. They initially tried various strategies such as imagining positive/negative emotions or looking at the location on the screen where they wanted the feedback signal to be. These strategies were not suitable for controlling the feedback signal. For most participants, learning was evident only after we suggested the use of potential regulation strategies that were related to the functional roles of the SMA and the PHC, e.g. imagining dancing or navigating home, respectively (Fig. 3; strategy suggestions). This finding suggests that the ability to control the feedback signal is due to a feedback-guided search for a cognitive control strategy.

On closer inspection, however, explicit cognitive control strategies cannot entirely explain the neurofeedback learning in our study. The specific cognitive strategies that the participants used potentially only explain the activity increase in the respective ROIs, but they are not linked to activity decreases of the other ROI. For example, while it is known that motor imagery increases activity in the SMA (e.g. Guillot et al., 2009), it is unclear how such a strategy could at the same time decrease activity in the PHC. Likewise, while it is known that spatial navigation imagery increases activity in the PHC (e.g. O’Craven & Kanwisher, 2000), it is unclear how such a cognitive strategy could at the same time decrease SMA activity. Further, even after we suggested control strategies related to motor and navigation imagery, some participants found other unrelated strategies more effective. One participant even learned to control the feedback signal without any strategy suggestions.

The fact that we initially did not suggest control strategies allowed us to shed new light on the potential learning mechanisms involved in neurofeedback training. We found that cognitive control strategies strongly facilitate neurofeedback learning. However, a model of neurofeedback learning that rests exclusively on explicit cognitive processing cannot entirely explain our results, e.g. it cannot explain how bidirectional control of the ROIs was achieved. Other feedback studies using electroencephalography (EEG) or physiological signals like heart-rate found evidence for the involvement of explicit cognitive control strategies, but also of more implicit operant conditioning based on reinforcement provided by the feedback (Dunn, Gillig, Ponsor, Weil, & Utz, 1986; Kotchoubey, Kubler, Strehl, Flor, & Birbaumer, 2002; Lacroix & Roberts, 1978; Neumann, Kubler, Kaiser, Hinterberger, & Birbaumer, 2003; Roberts, Birbaumer, Rockstroh, Lutzenberger, & Elbert, 1989; Roberts, Williams, Marlin, Farrell, & Imiolo, 1984; Schober & Lacroix, 1986; Siniatchkin, Kropp, & Gerber, 2000; Utz, 1987, 1994). Operant conditioning based neurofeedback has even been used to train volitional control of the activity of single neurons in animals (Fetz, 1969, 2007; Olds, 1965). However, because of the differences between these methods and real-time fMRI-based neurofeedback, it remains unclear if these findings can be generalized. Despite the lack of understanding of how real-time fMRI-based neurofeedback learning is accomplished, participants are usually given explicit control strategies. Only few studies in the field have used an operant conditioning approach (Bray et al., 2007), or have successfully trained participants without having suggested cognitive control strategies (Shibata et al., 2011). Our results thus indicate that suggesting explicit control strategies may initially facilitate learning, but that they are not necessary (Birbaumer et al., 2013; Shibata et al., 2011). Our results do not allow us to draw inferences about the need for such strategies during later stages of neurofeedback learning, or during the maintenance of learned self-regulation.

### 4.2 Behavioral effects of SMA and PHC control

After the neurofeedback training, we tested if exercising voluntary control over SMA and PHC led to ROI-specific performance changes. To test for behavioral consequences of regulating activity in the SMA, we asked the participants to perform a complex motor reaction time task. This task involved the planning and initiation of movements that require memorized, sequential, bilateral coordination; all of which are processes that are mediated by the SMA (Koeneke et al., 2004; Nachev et al., 2008; Tanji, 2001). The SMA has dense cortico-cortical connections with primary motor cortex (Johansen-Berg et al., 2004; Luppino, Matelli, Camarda, & Rizzolatti, 1993; Tokuno & Nambu, 2000), and it has been shown that activity in the SMA can modulate the cortical excitability of primary motor cortex (Arai, Lu, Ugawa, & Ziemann, 2012; Arai et al., 2011; Grefkes et al., 2008; Hamada et al., 2009; Matsumoto et al., 2007; Matsunaga et al., 2005; Shirota et al., 2012). We therefore hypothesized that voluntarily increasing activity in the SMA will lead to reduced reaction times. Our results partly confirmed this hypothesis. While we found no significant difference in reaction times between SMA_up_/PHC_down_ blocks compared to SMA_down_/PHC_up_ blocks, we found a significant negative correlation between self-regulated activity in the SMA and reaction times (Fig. 8). The correlation we found was specific to the initiation of the movement, but self-regulation of SMA activity did not affect the execution (i.e. the time it took to perform the finger sequence). This might be due to the fact that participants practiced the motor sequence extensively, and that during the production of highly practiced sequences, the SMA (and pre-SMA, which was partly included in our ROI) is especially important in the retrieval and initiation of sequences (Johansen-Berg et al., 2004; Kennerley, Sakai, & Rushworth, 2004; Tanji & Shima, 1994; Wiestler & Diedrichsen, 2013; Yanaka, Saito, Uchiyama, & Sadato, 2010).

Previous studies that successfully trained participants control over SMA activity did not test for behavioral effects of self-regulation (Hampson et al., 2011; Johnson et al., 2012). Amongst the studies that trained participants control over primary motor cortex (Bray et al., 2007; Chiew et al., 2012; deCharms et al., 2004; Yoo et al., 2008) only two reported behavioral effects. One study found that increased primary motor cortex activity caused a decrease in reaction times (Bray et al., 2007), and the other study did not find changes in reaction times after compared to before neurofeedback training of the primary motor cortex (Chiew et al., 2012). Instead of measuring reaction time differences between pre- vs. post-training of primary motor cortex, we measured reaction times while participants were actively up- and down-regulating activity in the SMA, i.e. participants were applying their newly learned self-regulation skill.

To test for behavioral consequences of regulating activity in the PHC, we presented words while participants regulated activity in their PHC. Afterwards, memory for these words was tested in an unexpected word memory test. The PHC is the principal neocortical input pathway to the hippocampus and is involved in memory formation. It has been shown that greater stimulus-evoked activity in the PHC correlates with better memory for scenes (Brewer et al., 1998; Gabrieli et al., 1997; Stern et al., 1996) and for words (Fernandez et al., 1999). Even more important for the present study, it has been shown that pre-stimulus PHC activity predicts memory for scenes (Turk-Browne et al., 2006) and words (Otten et al., 2006; Wagner et al., 1998), i.e. greater pre-stimulus activity is associated with better memory. We therefore hypothesized that voluntarily increasing activity in the PHC during word encoding will lead to improved memory for words. Our results, however, showed the opposite. Voluntarily increasing activity in the PHC led to decreased memory for words (Fig. 9).

This result is in stark contrast to the above mentioned studies which found that increased PHC activity correlated with better memory formation. However, similar to our results, a recent study also reported that decreased PHC activity led to better memory formation (Yoo et al., 2012). Using real-time fMRI, Yoo and colleagues monitored spontaneous fluctuations of PHC activity and triggered a memory probe depending on the activity level of the PHC. They found that memory probes that were triggered during decreased PHC activity levels were remembered significantly better than memory probes that were triggered during high PHC activity levels. They speculated that the lower levels of spontaneous PHC activity might reflect less processing, thus leaving more resources available for memory encoding. Likewise, our results could arise from a similar mechanism; voluntarily decreasing PHC activity could leave more resources available for memory encoding. Whereas Yoo et al. studied spontaneous fluctuations in PHC activity, the participants in our study voluntarily modulated PHC activity. It is thus possible that the cognitive strategy for up-regulating PHC activity competed for the same limited resources with memory encoding processes. For example, occupying the PHC with a spatial navigation strategy might withdraw resources important for verbal encoding. Such dual task interferences between self-regulation of brain activity and behavioral tasks have also been reported in other neurofeedback studies (Lutzenberger, Roberts, & Birbaumer, 1993; Rota et al., 2009). However, the participants in our study used comparable cognitive imagery in up- and down-regulation conditions (with respect to complexity and attentional demands). Hence, the interference that might underlie the present findings is related to neurofeedback-induced changes in specific neuronal populations of the PHC, rather than unspecific factors such as arousal or attention (Klingberg & Roland, 1997). Please note that we cannot completely exclude the possibility that the memory-related effects are partly due to activity changes in the SMA. However, given the functional specialization of our ROIs, such an explanation is unlikely.

## 5 Conclusion

Previous studies revealed that sustained brain activity that is not related to a particular stimulus or to task execution has an impact on cognitive function. Using a real-time fMRI neurofeedback approach, we extended this previous research by specifically training voluntary control over sustained brain activity to which we otherwise do not have conscious access to. Further, our novel experimental design allowed participants even to simultaneously learn bidirectional control over two functionally distinct brain regions. Control of these two regions caused characteristic behavioral effects that were related to the specific function of the brain region, i.e. reaction time changes were related to regulating activity in the SMA, and changes in memory performance were related to regulating activity in the PHC. Hence, the behavioral effects that we observed were due to changing regionally and functionally specific brain activity rather than to changes in general indices of cognitive efficiency such as attention or arousal. Because we did not test if the behavioral effects were also evident independent of actively self-regulating, we cannot conclude if there are lasting plastic changes that go beyond those related to temporarily increasing ongoing activity.

The sample size in our study was rather small and, as a consequence, the behavioral effects of particularly the reaction time task were somewhat statistically weak. Nonetheless, the faster responses when voluntarily increasing activity in the SMA might indicate the potential for cognitive enhancement through real-time fMRI-based brain training. Learning voluntary control of the SMA could also be used as a clinical treatment for Tourette's syndrome, where SMA activity is linked to motor tics (Bohlhalter et al., 2006; Hampson et al., 2011; Stern et al., 2000), or it could be used to promote recovery from stroke (Braun, Beurskens, Borm, Schack, & Wade, 2006; de Vries & Mulder, 2007; Sitaram et al., 2012). Also learning voluntary control over PHC activity might have important implications for a range of cognitive functions such as learning and memory (Asaka et al., 2005; Seager, Johnson, Chabot, Asaka, & Berry, 2002; Yoo et al., 2012), as well as neuropsychiatric conditions such as posttraumatic stress disorder (Geuze, Vermetten, Ruf, de Kloet, & Westenberg, 2008; Werner et al., 2008), schizophrenia (Bodnar et al., 2012; Diederen et al., 2010; Ragland et al., 2009), or Alzheimer's disease (Hyman, Van Hoesen, Damasio, & Barnes, 1984; Peters et al., 2009). However, in order to demonstrate the usefulness of this approach in clinical practice, follow-up patient studies with larger samples are needed.

Using neurofeedback to induce regionally specific changes in brain activity without drugs goes beyond conventional brain imaging studies that are only correlational. Similarly to other approaches that interfere with brain activity like transcranial magnetic stimulation, deep brain stimulation, neuropharmacological interventions, and brain lesions, it permits us to manipulate behavior causally.

## Figures and Tables

**Table 1:** Areas related to self-regulation (group whole brain analysis).

Areas de-/activated during self-regulation blocks (i.e. SMA_up_/PHC_down_ and SMA_down_/PHC_up_ blocks)					
Anatomical label	t-Value	De-/activation [−/+]	MNI coordinates		
			*x*	*y*	*z*
Bilateral middle cingulate cortex	12.77	+	−2/8	25	32
Left superior parietal lobe	12.42	+	−18	−70	42
Right superior frontal gyrus	12.40	+	26	3	58
Bilateral precuneus	10.86	+	−6/14	−55	10
Left parahippocampal gyrus	10.47	+	−30	−40	−10
Bilateral cerebellum (lobule VI)	8.67	+	−30/34	−64	−24
Bilateral inferior parietal cortex	8.62	+	−48/40	−71	28
Bilateral SMA	8.11	**+**	−2/10	−12	64
Right hippocampus	7.61	**+**	30	−36	−14
Left putamen	6.14	**+**	−24	−2	12
Bilateral superior occipital cortex	9.89	–	−22/24	−94	20
Bilateral ventral occipital cortex	8.98	**–**	−22/28	−86	−9
