# Supplementary material for: Manipulating motor performance and memory through real-time fMRI neurofeedback
Source: Biol Psychol. 2015 May;108:85–97. doi: 10.1016/j.biopsycho.2015.03.009 (PMC4433098; doi:10.1016/j.biopsycho.2015.03.009)
Supplement: Supplementary file 1 [file mmc1.doc]

**Supplemental Information**

**Manipulating motor performance and memory through real-time fMRI neurofeedback**

Frank Scharnowski, Ralf Veit, Regine Zopf, Petra Studer, Simon Bock, Jörn Diedrichsen, Rainer Göbel, Klaus Mathiak, Niels Birbaumer, Nikolaus Weiskopf

Supplemental Figures

[Figure S1 Neurofeedback learning curve.](#__RefHeading___Toc297930650) 2

[Figure S2 Whole brain analyses. 3](#__RefHeading___Toc297930651)

Supplemental Table

[Table S1 Areas related to self-regulation. 4](#__RefHeading___Toc297930652)


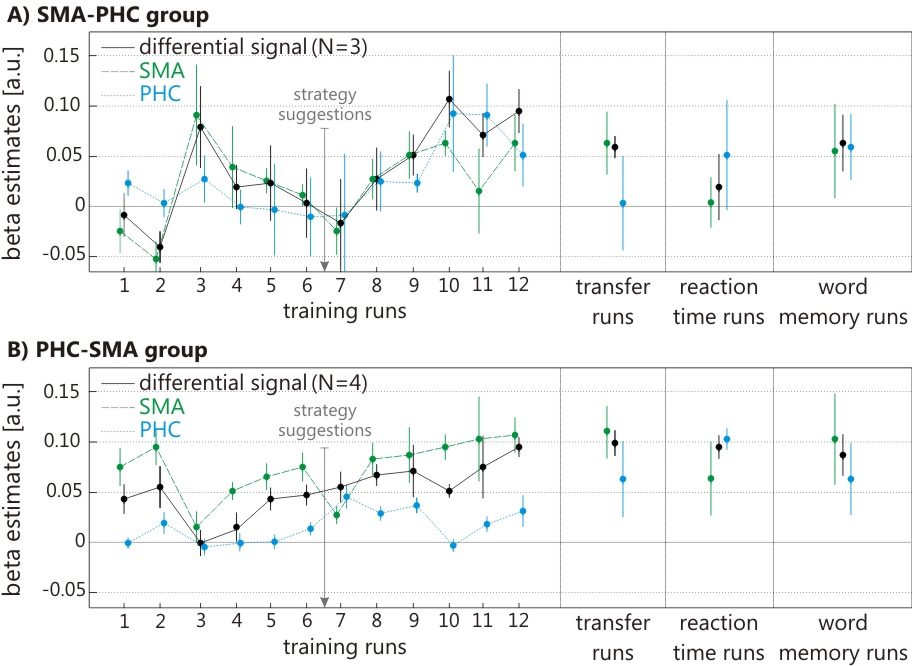


**Figure S1: Neurofeedback learning curve separately for the participants that received (A) SMA-PHC and (B) PHC-SMA feedback.**

Learning to control the differential feedback signal did not depend on the type of feedback signal, i.e. SMA-PHC or PHC-SMA. (A) The three participants that received SMA-PHC feedback learned to control the feedback signal (differential signal linear regression: r^2^ = 0.43, F(1,10) = 7.64, p = 0.02), and subsequently maintained that control during transfer runs (transfer run column; differential signal 1-sample t-test: t(2) = 6.92, p = 0.02). (B) Also the four participants that received PHC-SMA feedback learned to control the feedback signal (differential signal linear regression: r^2^ = 0.49, F(1,10) = 9.71, p = 0.01), and subsequently maintained that control during transfer runs (transfer run column; differential signal 1-sample t-test: t(3) = 7.30, p < 0.01). There was no difference in control over the differential feedback signal between the SMA-PHC and the PHC-SMA groups during the transfer runs (paired t-test: t(5) = -1.69, p = 0.16). After the 6^th^ training run, specific regulation strategies related to motor imagery and spatial navigation were suggested to the participants. Error bars represent one standard error of the mean.

A) Areas activated during SMA_up_/PHC_down_ blocks


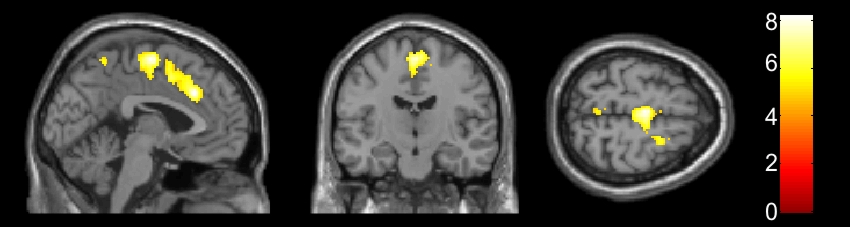


B) Areas activated during SMA_down_/PHC_up_ blocks

**
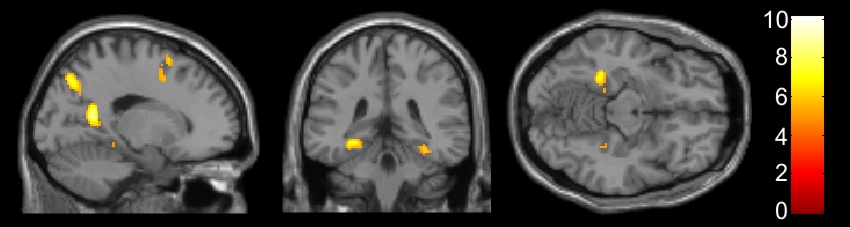
**

**Figure S2: Whole brain analyses.**

Shown are brain activation maps for (A) SMA_up_/PHC_down_ blocks, and for (B) SMA_down_/PHC_up_ blocks. (A) During SMA_up_/PHC_down_ blocks, activity was increased in the anterior/middle cingulate gyrus bilaterally, the SMA bilaterally (overlapping with the trained ROI), the right inferior frontal gyrus (not shown), the left middle frontal gyrus (not shown), the insula bilaterally (not shown), and the right cerebellum (not shown); activity decreased in the left/middle superior visual cortex (not shown). (B) During SMA_down_/PHC_up_ blocks, activity was increased in the precuneus bilaterally, the left parahippocampal gyrus (overlapping with the trained ROI), the left superior parietal lobe, the right inferior parietal lobe, the superior/middle frontal gyrus, the right (para-)hippocampus, and the right middle cingulate cortex (not shown); activity decreased in the left superior medial gyrus, the superior occipital cortex bilaterally, and the right primary visual cortex (all not shown). The figures show contrast maps thresholded at p < 0.05 (corrected for multiple comparison using FWE) on the MNI template brain. For details, see Supplementary Table S1.

| Table S1. Areas related to self-regulation (group whole brain analysis) | | | | | | | | | |  | | | |
| --- | --- | --- | --- | --- | --- | --- | --- | --- | --- | --- | --- | --- | --- |
|  |  | | | | | | | | |  | | | |
| A) Areas de-/activated during SMA_up_/PHC_down_ blocks | | | | | | | | | |  | | | |
| **Anatomical label** | | **t-value** | | **de-/activation** | **MNI coordinates** | | | | |  | | | |
|  | |  | | **[ - / + ]** | **x** | **y** | | **z** | |  | | | |
| Bilateral anterior/middle cingulate gyrus | | 8.20 | | + | -2/8 | 22 | | 36 | |  | | | |
| Bilateral SMA | | 8.03 | | + | -2/2 | -12 | | 64 | |  | | | |
| Right inferior frontal gyrus | | 7.46 | | + | 54 | 26 | | 2 | |  | | | |
| Left middle frontal gyrus | | 6.22 | | + | -28 | 12 | | 46 | |  | | | |
| Bilateral insula | | 6.11 | | + | -38/40 | 20 | | 5 | |  | | | |
| Right cerebellum (Lobule VI & VIIa) | | 5.77 | | + | 22 | -78 | | -20 | |  | | | |
| Left superior parietal cortex | | 5.49 | | + | -2 | -54 | | 62 | |  | | | |
| Left middle/superior occipital cortex | | 5.97 | | - | -12 | -97 | | 8 | |  | | | |
|  | |  | |  | | | | |  | |  |  |  |
| B) Areas de-/activated during SMA_down_/PHC_up_ blocks | | | | | | | | | |  | | | |
| **Anatomical label** | | **t-value** | | **de-/activation** | **MNI coordinates** | | | | |  | | | |
|  | |  | | **[ - / + ]** | **x** | | **y** | **z** | |  | | | |
| Bilateral precuneus | | 9.40 | | + | --8/16 | | -52 | 12 | |  | | | |
| Left parahippocampal gyrus | | 8.39 | | + | -28 | | -40 | -10 | |  | | | |
| Left superior parietal lobe | | 8.39 | | + | 18 | | -74 | 44 | |  | | | |
| Right inferior parietal cortex | | 7.47 | | + | 44 | | -68 | 22 | |  | | | |
| Bilateral superior/middle frontal gyrus | | 7.11 | | + | -26/28 | | 6 | 59 | |  | | | |
| Right (para-)hippocampus | | 6.84 | | + | 32 | | -36 | -16 | |  | | | |
| Right middle cingulate cortex | | 5.50 | | + | 4 | | 24 | 34 | |  | | | |
| Left superior medial gyrus | | 7.30 | | - | -10 | | 66 | 18 | |  | | | |
| Bilateral superior occipital gyrus | | 6.03 | | - | -22/24 | | -94 | 21 | |  | | | |
| Right cuneus | | 5.76 | | - | 14 | | -98 | 6 | |  | | | |
|  | |  |  | |  | |  |  | |  | | | |
